# Supplementary material for: Intraoperative phrenic nerve stimulation to prevent diaphragm fiber weakness during thoracic surgery
Source: PLoS One. 2025 Apr 1;20(4):e0320936. doi: 10.1371/journal.pone.0320936 (PMC11961012; doi:10.1371/journal.pone.0320936)
Supplement: Table S1 — (DOCX) [file pone.0320936.s005.docx]

**Table S1. Serious adverse events in study participants.** While 14 of 21 experienced a serious adverse event, all were determined to be expected consequences of open cardiothoracic surgery. None was found to be related specifically to study participation.

|  |  |  |  |
| --- | --- | --- | --- |
|  | **Total** | **Affected / at Risk (%)** | **# Events** |
|  |  | 14/21 (66.67%) |  |
|  | Arrhythmias | 10/21 (47.62%) | 11 |
|  |  |  |  |
|  | Anemia | 3/21 (14.29%) | 4 |
|  |  |  |  |
|  | Dysphagia | 3/21 (14.29%) | 3 |
|  |  |  |  |
|  | Acute respiratory failure | 2/21 (9.52%) | 2 |
|  |  |  |  |
|  | Methycillin sensitive staph aureus infection | 2/21 (9.52%) | 2 |
|  |  |  |  |
|  | Acute Kidney Injury | 1/21 (4.76%) | 1 |
|  |  |  |  |
|  | Acute respiratory insufficiency | 1/21 (4.76%) | 1 |
|  |  |  |  |
|  | Near-syncope | 1/21 (4.76%) | 1 |
|  |  |  |  |
|  | Low cardiac index | 1/21 (4.76%) | 1 |
|  |  |  |  |
|  | Sternal wound infection | 1/21 (4.76%) | 1 |
|  |  |  |  |
|  | Tachycardia | 1/21 (4.76%) | 1 |
|  |  |  |  |
|  | Acute MCA stroke | 1/21 (4.76%) | 1 |
|  |  |  |  |
|  | Pulmonary embolism | 1/21 (4.76%) | 1 |
|  |  |  |  |
|  | Urinary Tract Infection | 1/21 (4.76%) | 1 |
|  |  |  |  |
|  | Sternal malunion | 1/21 (4.76%) | 1 |
|  |  |  |  |
|  | Cellulitis | 1/21 (4.76%) | 1 |
|  |  |  |  |
