# Supplementary material for: Intraoperative phrenic nerve stimulation to prevent diaphragm fiber weakness during thoracic surgery
Source: PLoS One. 2025 Apr 1;20(4):e0320936. doi: 10.1371/journal.pone.0320936 (PMC11961012; doi:10.1371/journal.pone.0320936)
Supplement: Table S2 — (DOCX) [file pone.0320936.s006.docx]

**Table S2. Nonserious adverse events in study participants.** The threshold for reporting other adverse events was 5%. While every participant experienced a nonserious adverse event, all were determined to be expected consequences of open cardiothoracic surgery. None was found to be related specifically to study participation.

|  |  |  |  |
| --- | --- | --- | --- |
|  | **Total** | **Affected / at Risk (%)** | **# Events** |
|  |  | 21/21 (100%) |  |
|  | Postoperative Pain | 20/21 (95.24%) | 20 |
|  |  |  |  |
|  | Anemia | 17/21 (80.95%) | 17 |
|  |  |  |  |
|  | Pleural effusion | 10/21 (47.62%) | 10 |
|  |  |  |  |
|  | Hyperglycemia | 10/21 (47.62%) | 10 |
|  |  |  |  |
|  | Atelectasis | 9/21 (42.86%) | 9 |
|  |  |  |  |
|  | Leukocytosis | 6/21 (28.57%) | 6 |
|  |  |  |  |
|  | Acute kidney injury | 4/21 (19.05%) | 4 |
|  |  |  |  |
|  | Hypotension | 4/21 (19.05%) | 4 |
|  |  |  |  |
|  | Bradycardia | 4/21 (19.05%) | 4 |
|  |  |  |  |
|  | Thrombocytopenia | 3/21 (14.29%) | 4 |
|  |  |  |  |
|  | Pericardial effusion | 2/21 (9.52%) | 2 |
|  |  |  |  |
|  | Pulmonary edema | 2/21 (9.52%) | 2 |
|  |  |  |  |
|  | Pneumonia | 2/21 (9.52%) | 2 |
|  |  |  |  |
|  | Bilateral lower extremity edema | 2/21 (9.52%) | 2 |
|  |  |  |  |
