# Supplementary material for: Intraoperative phrenic nerve stimulation to prevent diaphragm fiber weakness during thoracic surgery
Source: PLoS One. 2025 Apr 1;20(4):e0320936. doi: 10.1371/journal.pone.0320936 (PMC11961012; doi:10.1371/journal.pone.0320936)
Supplement: S2 File — (PDF) [file pone.0320936.s008.pdf]

Protocol

1. Project Title:

The effect of hemidiaphragm stimulation during surgery on gene expression and single fiber contractile properties in humans

2. Investigator(s):

Barbara Smith, PhD, PT;
Thomas Beaver, MD, MPH

3. Abstract:

Approximately 10% of the patients who receive mechanical ventilation (MV) experience difficulty weaning, and these patients account for approximately 40-50% of the total MV days. Numerous animal studies have documented that MV induced muscle inactivity leads to diaphragm atrophy, oxidative stress, altered diaphragm gene expression and reduced muscle strength in as little as 6-24 hours. This phenomenon has been termed ventilator induced diaphragm dysfunction (VIDD). Spontaneous breathing for 5 minutes every 6 hours over a 24 hour period of MV support can attenuate VIDD by approximately 50% in a rodent model, indicating that very brief periods of normal diaphragm activity can be protective. Recent human work has documented VIDD (diaphragm muscle fibers atrophying ~ 55%) following approximately 39 hours of controlled MV. Clinical human studies have shown that patients experiencing difficulty weaning from MV have impaired diaphragm performance, consistent with changes seen in VIDD models. Therefore, while there is growing evidence that MV rapidly leads to VIDD in animals and humans, and brief periods of diaphragm activity during MV support can lessen VIDD in a rodent model, there are no data examining strategies to attenuate this problem in humans. We have data examining human diaphragm gene expression (within subjects design) at the beginning and end of ~5 hours of surgery/MV support. The data revealed up regulation of several diaphragm genes controlling ubiquitin mediated catabolic activity and down regulation of genes critical to normal muscle contractile function. We propose to study the effects of periodically stimulating one hemidiaphragm and using the unstimulated hemidiaphragm as a control in a human MV/surgical model. The right or left hemidiaphragm will be stimulated via the corresponding phrenic nerve during surgery depending upon the ease of access. Costal diaphragm muscle samples will be obtained from each hemidiaphragm at the end of surgery. Gene expression, mitochondrial function and single muscle fiber contractile properties in the stimulated and unstimulated hemidiaphragms will be contrasted. This study will provide the first data examining the molecular and contractile effects of increased diaphragm activity during periods of MV/surgery in humans. The results from this research study will help direct efforts to develop rehabilitation interventions in the preoperative and postoperative period aimed at preventing VIDD in humans.

4. Background:

Failure to wean from mechanical ventilation (MV) is a growing clinical and economic problem in the United States health care system. The vast majority of patients supported by MV are quickly weaned, but a minority (~10%) of these patients experience difficult weaning and require prolonged MV support, often for months. Approximately 250,000 patients annually in the US experience difficult weaning and the estimated annual cost of providing care during these prolonged MV support episodes is 16 billion dollars [1].

Several articles have documented that patients experiencing difficult weaning have inactivity-induced diaphragm muscle dysfunction [2-6]. It is not surprising that the use of MV leads to muscle atrophy and dysfunction. Mechanical ventilators are in essence, artificial breathing “muscles” that pump air into the patients’ lungs, facilitating gas exchange. While MV support is life-saving for patients with respiratory failure, MV support can have deleterious effects on the respiratory muscles. Numerous [7-17] animal studies have shown that even short periods of controlled MV support (6-24 hours) can lead to physiologically significant diaphragm atrophy, decreased muscle force production, oxidative stress and gene expression changes promoting muscle dysfunction which has been termed ventilator induced diaphragm dysfunction (VIDD). Furthermore, animal work has shown that the diaphragm atrophies approximately 8 times faster than limb muscle, indicating that the diaphragm is uniquely sensitive to inactivity-induced atrophy and dysfunction [10]. While rodent diaphragm function is extraordinarily sensitive to reduced activity, it takes very little activity to maintain function. Gayan-Ramirez [18] has shown that allowing rats to breathe spontaneously for 5 minutes every 6 hours reduces the VIDD effect by 50% when animals receive 24 hours of MV support. Sassoon [19] found that ventilating rats with assist–control ventilation, which requires the animals to generate some diaphragmatic activity, also attenuated VIDD when compared to controlled MV, which eliminates all active diagram activity. Increased activity is not the only possible means of attenuating VIDD. Maes et al. [20] found that leupeptin, an inhibitor of lysosomal proteases and calpain completely prevented VIDD and atrophy over a 24 our period in a rat MV model. Trolox can also partially protect the diaphragm from VIDD by decreasing myofilament protein substrate availability to proteasomes in rats [7]. Data examining the human diaphragm following periods of MV are sparse, but largely consistent with the animal data.
Recent work by Levine, et al. in humans has shown that controlled MV for ~ 36 hours led to 55% atrophy of diaphragm muscle fibers, increased markers of oxidative stress and upregulation of genes controlling pathways leading to muscle atrophy [21]. Like animals, the rate of diaphragm atrophy in humans is much greater than seen in limb muscles. For example, 5 weeks of bedrest inactivity led to 12% atrophy in the thigh and calf muscles in healthy young subjects [22]. Ohira reported that 120 days of bedrest in healthy young subjects resulted in a 32% reduction in the soleus muscle cross sectional area [23]. Buckey found that 10 days of bedrest atrophied calf muscles by 7% [24]. Ogawa found that 20 days of inactivity reduced quadriceps muscle cross sectional area by 6% [25]. Clearly, the human diaphragm atrophies much faster than human limb muscle. Despite the importance of VIDD’s probable contribution to weaning difficulties, the vast majority of work examining the effects of MV and VIDD has been conducted in

animal models and has focused on the dysfunction side of the muscle plasticity continuum, with sparse attention given to measures to reduce VIDD [9-12, 15, 17, 26]. Only two studies have directly studied the impact of MV on human diaphragm structure and none have directly measured muscle contractile performance following periods of MV. Levine's recent report on the effects of 36 hours of MV in humans was discussed earlier. The only other human study used a case control design that qualitatively showed profound diaphragm muscle fiber atrophy in a young child ventilated for 47 days compared to a child ventilated for 3 days [27]. The current evidence indicates that short periods of MV leads to VIDD in animal models and difficult to wean humans clinically demonstrate VIDD. Given the evidence of VIDD, it is clear that mechanistic human data are needed to design strategies to prevent or treat the problem.

In this study, we propose to stimulate either the right or left human hemidiaphragm hourly during routine cardiothoracic (CT) surgery and to obtain diaphragm muscle biopsies from the stimulated and unstimulated hemidiaphragms at the end of surgery for analysis with micro gene arrays, mitochondrial function and single muscle fiber contractile properties. To our knowledge, this project would provide the first simultaneous data on the human diaphragm's gene expression, mitochondrial function and contractile responses to activity during surgery and MV support. This knowledge will be invaluable to developing strategies to protect the diaphragm from the deleterious effects of MV and surgery.

This project will help establish our human diaphragm surgery/MV model, facilitating applications for support from the NIH to study preoperative diaphragm training paradigms and intraoperative pharmacologic interventions.

5. Specific Aims:

Aim 1: Compare the expression of the human diaphragm genes that control energy metabolism, excitation–contraction coupling, contractile proteins and oxidative stress in stimulated and unstimulated diaphragm muscle samples.

Aim 2: Compare the single muscle fiber contractile characteristics (subdivided by muscle fiber type) of stimulated and control diaphragm muscle fibers.

Aim 3: Compare the mitochondrial function of stimulated and control diaphragm muscle fibers.

We hypothesize that intermittent stimulation of either the right or left human hemidiaphragm during thoracic surgical procedures and lung transplant lasting approximately 5 hours or longer will prevent or attenuate inactivity-induced changes in gene expression, mitochondrial function and single fiber contractile properties as compared to the unstimulated hemidiaphragm.

6. Research Plan:

Subjects:

Patients undergoing regularly scheduled cardiothoracic surgery, including lung transplants, at Shands Hospital at the University of Florida will be recruited to enroll in this prospective study. From each subject an informed consent will be obtained to donate two diaphragm muscle biopsy samples during the surgery.

After recruitment, an initial evaluation consisting of basic demographic information as well as diagnostic information pertaining to the surgery will be performed before the scheduled surgery to search for exclusion factors. In addition, we will track medications, ventilator use, complications, and length of stay during the patient's hospitalization for surgery. Up to 100 subjects will be studied.

Exclusion Criteria:

1. Age < 18 or > 80,
2. history of prior surgery to the diaphragm or pleura;
3. a diagnosis of COPD will be determined from a clinical history consistent with chronic bronchitis and/or emphysema, a long history of cigarette smoking, and pulmonary function tests consistent with irreversible airflow obstruction (FEV1 < 40% predicted, according to European Respiratory Society criteria (1).
4. a diagnosis of chronic heart failure (NYHA class IV) (2)
5. clinical diagnosis of other lung disease (cystic fibrosis, bronchiectasis, lung cancer; etc.);
6. renal insufficiency (serum creatinine > 1.6 mg/dl);
7. severe hepatic disease (any liver function tests > 1.5 times the upper limit of normal);
8. undernourishment (body mass index < 20 kg/m^2),
9. chronic uncontrolled or poorly controlled metabolic diseases (e.g., diabetes, hypo- or hyperthyroidism)
10. orthopedic diseases, suspected paraneoplastic or myopathic syndromes,
11. if in the surgeons' judgment the patients' clinical status warrants, diaphragm stimulation will be stopped and biopsies will not be obtained,
12. items #3 and #5 above will not apply to transplant patients.

Experimental Procedure:

Before the surgery:

A pulmonary function test will be performed to determine whether the subject qualifies for the study.

PFT: Subjects will complete a forced expiratory maneuver to determine eligibility for training. Tests will be completed according to American Thoracic Society guidelines,(3) and 3 to 5 trials administered, separated by 2 minutes of rest. If an individual demonstrates FEV1<40% of predicted, he or she will be removed from any further

participation in the study. An $FEV_{1.0}$ of 40% or less of predicted as an exlcusionary factor will not apply to transplant patients.

In addition, the qualified subjects will undergo maximum inspiratory pressure test before the scheduled surgery.

Maximal Inspiratory Pressure: Subjects will perform a maximal inspiratory pressure maneuver from residual volume, in accordance with American Thoracic Society testing guidelines.(3) Trials will be administered until 5% variability is achieved between three trials (typically achieved within 5 trials), and separated by a two-minute rest. A short rest between maneuvers will be provided to prevent muscle fatigue.

In conjunction with respiratory testing, we will monitor baseline and exertional blood pressure, heart and respiratory rate, and pulse oximetry. The following tests will be completed on all subjects.

During t surgery following procedures will be performed,

Diaphragm stimulation: Either the right or the left hemidiaphragm will be stimulated during routine cardiothoracic surgical procedures. This will be decided by the surgeon on the basis of ease of access to the corresponding phrenic nerve. Rodent models of MV have shown that different neuromuscular blockers can exert varied effects on the diaphragm contractile properties (4-7). The right or the left hemidiaphragm will be stimulated via the corresponding phrenic nerve with a temporary cardiac pacer (Medtronic 5388) with wire electrodes. The surgeon will identify the phrenic nerve on the pericardium and the pacing electrodes will be sutured to the pericardium immediately adjacent (~8 mm) to either side of the phrenic nerve (8). It is not necessary for the pacing electrodes to touch the phrenic nerve to stimulate the phrenic nerve and generate a hemidiaphragm contraction.

Dr. Beaver has observed vigorous left hemidiaphragm contractions on many occasions during temporary heart pacing when electrodes were applied to the pericardium close to the left phrenic nerve. Great care will be taken to ensure that the stimulating electrodes do not contact the phrenic nerve. The left phrenic nerve will be stimulated near the pulmonary artery to prevent pacing the heart. The right phrenic nerve will be stimulated on the right side of the pericardium.

Patients receive non-depolarizing neuromuscular blockers during surgery to prevent limb muscle contractions. The diaphragm is more resistant to neuromuscular blockade than limb muscles, and remains responsive to stimulation despite neuromuscular blockade sufficient to temporarily paralyze limb muscles (9, 10). The surgeon will select the hemidiaphragm for stimulation depending upon the ease of access to the corresponding phrenic nerve. There is no evidence that the muscle fiber contractile characteristics or gene expression differ between the right and left human hemidiaphragms.

Once the electrodes are sutured in place, the stimulator will be activated with the lowest possible current. The stimulator current will then be systematically increased until a vigorous contraction of the corresponding hemidiaphragm is observed, which will be

termed the threshold stimulus. The stimulus intensity will be adjusted to three times the threshold level for subsequent stimulation. The hemidiaphragm on the unstimulated side will be observed to verify that it is not entrained with the hemidiaphragm contractions on the stimulated side. Stimulation will be conducted for one minute (30 pulses per minute, 15 msec duration) either 30 or 60 minutes following initiation of MV and every 30 or 60 minutes thereafter. The frequency of stimulation (every 30 or 60 minutes) will be determined by the estimated duration of surgery. In order for both human hemidiaphragms to generate contractions, stimulation must be applied to both phrenic nerves or hemidiaphragms (11). The temporary wire electrodes that are used for the electrical stimulation will be removed at the end of the surgery.

Our model is not a pure MV inactivity paradigm with the addition of surgery, but it is not possible to ventilate humans and obtain diaphragm biopsies for experimental purposes, as is possible with animal models. Our surgery/MV model, however, is the actual clinical situation that often leads to VIDD and difficult weaning in humans, and thus, our results will be highly relevant to the clinical management of these patients.

Data safety monitoring board:

A data safety monitoring board comprised of three physicians will be impaneled before any additional subjects are studied. Prior to collecting additional data, the panel will be convened to review the study protocol, safety measures and establish operating policies. The data safety monitoring board will meet at least annually and will be given immediate reports of any serious and unexpected adverse events that may occur. DSMB findings and reports the will be included in the annual renewal of this protocol to the IRB.

Diaphragm Biopsies:

The method selected to obtained diaphragm biopsies was designed to minimize the risk of bleeding, phrenic nerve injury, or diaphragmatic impairment. The surgeon will obtain a full-thickness diaphragm sample, approximately 6 mm X 20 mm, from the antero-lateral coastal regions of the stimulated and unstimulated hemidiaphragms, shortly before the surgery is completed. By harvesting the diaphragm biopsy from the anterior costal diaphragm, in a region opposite of the phrenic nerve insertion, we will minimize the proximity to phrenic insertion. The wounds will be closed with sutures. The surgeon will place the biopsy samples on ice and hand them to study staff. Tissue samples will be divided using the procedures described below. One part of the sample will be sent to Christiaan Leeuwenburgh's laboratory for mitochondrial respiration and gene analysis. One sample will be sent to Dr. Karan Esser's laboratory (University of Florida, College of Medicine) for RNA analysis. Samples to be analyzed with micro gene array and RNA analysis will be immediately placed in RNAlater solution and stored at –80° C until analysis. Another part of the sample will be sent to Dr Leo Ferreira's laboratory (College of Health and Human Performance, University of Florida) for contractile and fiber type measures. Samples destined for single fiber contractile properties will be placed in a relaxing solution containing 50% glycerol (vol/vol) and stored at 20°C for no more than 4 wk before subsequent analysis. (12) A portion of the tissue samples will be sent to a non-UF investigator, Dr. Coen Ottenheijm at the University of Arizona for

analysis of muscle titan integrity. After harvest, tissue samples will be coded to protect subject confidentiality and tissue samples will not contain identifying information such as the subject name, medical record are other identifying information. All of the tissue will be consumed during the analysis of the various variables and will not be stored or bank for use outside of this protocol.

In addition we will request the subject's permission to be videotaped for this research project. The process of stimulation of the phrenic nerve and the contraction or movement of the diaphragm muscle will be videotaped during the surgery. A separate consent [Consent to be videotaped and to different uses of the videotape(s)] will be obtained from the subjects.

C. After Surgery:

Data Analysis:

Microarray Analysis:

The gene micro array analysis will be performed in Dr Christians Leeuwenburgh's laboratory (Department of Aging at the University of Florida, College of Medicine).
In brief, a U133 plus 2.0 Affymetrix GeneChip or similar product will be used to query the entire human genome (~ 54,000 probes). Total RNA will be harvested for cDNA preparation using protocols and reagents recommended by Qiagen Inc. cDNA will be prepared using protocols recommended by Affymetrix and reagents obtained from Invitrogen. Biotin labeled-cRNA preparation will be accomplished using protocols and reagents recommended by Affymetrix. The labeled-cRNA will be used to interrogate DNA microarrays prepared by Affymetrix. cRNA will be synthesized from cellular RNA from specimens based on protocols and recommendations from the GeneChip manufacturer, Affymetrix.

Single fiber contractile and fiber type measures:

Single fiber contractile and fiber type measures will be done in Dr Ferreira's laboratory (Physiology laboratory at the Center for Exercise Science, University of Florida). Dr Ferraria's laboratory has extensive experience with these techniques (13).

Muscle fibers treatment:

Following collection, muscle fiber bundles will be stretched ~20%, pinned on cork, and placed in a relaxing solution consisting of 59.0 mM potassium acetate, 6.7 mM magnesium acetate, 5.6 mM NaATP, 10 mM EGTA, 2.0 mM dithiothreitol, 15.0 mM creatine phosphate, 1 mg/ml phosphocreatine kinase, and 50 mM imidazole for a total ionic strength of 200 mM at a pH of 7.0 at 5°C and then shipped to Dr Mantilla's laboratory for analysis. The fiber bundles can be stored in relaxing solution for up to 3 weeks before analysis.

Myosin heavy chain concentration and single-fiber gel electrophoresis:

Single fiber segments (~1.5-2.5 mm in length) will be fixed in 4% paraformaldehyde for 30 s and placed on a microscope stage with a CCD camera. The fiber image will be

projected onto a video screen, and the total number of sarcomeres will be counted. Fibers will be placed in 25 µl of SDS sample buffer containing 62.5 mM Tris-HCl, 2% (wt/vol) SDS, 10% (vol/vol) glycerol, 5% 2-mercaptoethanol, and 0.001% (wt/vol) bromophenol blue (pH 6.8). Boiling for 2 min will denature the samples. Samples will be separated electrophoretically in gradient gels (stacking gel: 3.5% acrylamide -pH 6.8, and separating gel: 5-8% acrylamide -pH 8.8), and gels will be silver stained. Control samples at known concentrations of myosin heavy chain will be run for quantification. Myosin heavy chain concentration is determined by dividing the myosin heavy chain content determined from the myosin standard curve by the fiber volume. This concentration will then multiplied by the half-sarcomere volume of the fiber, normalized to optimal length (2.5 µm/sarcomere). Space limitations prevent complete descriptions of the procedures for measuring myosin heavy chain concentrations and gel electrophoresis for fiber classification, but more details on these methods can be found in articles from Dr. Mantilla's laboratory.(14, 15)

Single-fiber mechanical measurements:

Fibers will be retrieved from the relaxing solution and fiber bundles will be placed in a solution containing 1% Triton X-100 to permeabilize the plasma membrane for ~20 min, and dissected under a dissecting microscope. Single fibers will be transferred from the skinning solution to a relaxing solution (pCa 9.0) for force measurements. Force measurements will be performed in solutions containing the following (in mM): 10.0 EGTA, 1.0 free Mg^{2+}, 5.0 MgATP, 15.0 creatine phosphate, 50.0 imidazole, 2.0 DTT, and PCK at 1 mg/ml with a total ionic strength of 150 mM. The relaxing solution will have pCa of 9.0, and the activating solution a pCa of 4.0. Fiber ends will be fixed by exposing them to a 5% glutaraldehyde solution to maintain noncompliant attachments of the fibers to a force transducer and servo-controlled motor. Fibers will be mounted horizontally on two small stainless steel hooks in a temperature-controlled flow-through acrylic chamber located on the stage of an inverted microscope. Fibers will be attached at one end to a force transducer (resonant frequency of 5 kHz), and the other end to a servo-motor (step time: 800 µs). Sarcomere length will be set at 2.5 µm and monitored by first-order laser diffraction. Signals will be recorded with a custom-designed LabView-based software and a data acquisition board. Fiber dimensions will be measured to calculate fiber cross-sectional area. A baseline force measurement will be obtained while fibers are perfused with a pCa 9.0 solution. The perfusate will be switched to a pCa 4.0 solution (in the same flow-through chamber) to maximally activate the fibers, and after maximal activation is achieved, the fiber will again be perfused with a pCa 9.0 solution to verify that force returns to its original baseline level. Maximum specific force (N/cm2) will be calculated by dividing the maximum isometric force by fiber cross-sectional area. Approximately 4 fibers of each fiber type will be analyzed. This technique has been previously reported in greater detail. (15)

Mitochondrial function measurements in diaphragm muscle:

Elevated levels of oxidative stress produced during mechanical ventilation (MV) directly contribute to diaphragmatic atrophy and contractile dysfunction, thus preventing the successful removal of patients from the ventilator (i.e. "weaning") (16, 17). Numerous lines of evidence (18, 19) have surfaced implicating reactive oxygen species (ROS) and

specifically mitochondrial-mediated signaling pathways in mitochondrial ventilation (MV)-induced diaphragmatic dysfunction.

Human biopsy pickups will be performed by specific personnel from Dr. Leeuwenburgh's lab. Diaphragm muscle tissue (~20-30 mg) will be immediately placed on ice in Buffer. The sample will be taken to Dr. Leeuwenburgh's laboratory, and samples will be blotted and dissected free of blood, fat, and connective tissue.

The muscle sample will then be separated into different portions and processed as follows:

~8 mg will be placed on ice in isolation medium (10 mM Ca-EGTA, 0.1 μM free calcium, 20 mM imidazole, 20 mM taurine, 49 mM K-MES, 3 mM K2HPO4, 9.5 mM MgCl2, 5.7 mM ATP, 15 mM phosphocreatine, 1 μM leupeptin, pH 7.1) and be utilized for respirometry; ~30 mg of muscle tissue will be frozen in liquid nitrogen, stored at -80°C (in Room P1-09), and used for BN-PAGE; remaining tissue will be frozen immediately in liquid nitrogen and stored at −80°C (Room P1-09) for further analysis (i.e. biochemical, immunoblotting, PCR).

Procedure

The parameters of mitochondrial function will be assessed in these patients using conventional biochemical (i.e., electrophoresis and immunoblotting) and histological (light and electron microscopy) methods, as well as several novel technologies listed below;

High-resolution respirometry will be utilized in freshly permeabilized human muscle samples to assess mitochondrial respiration, and electron transport chain and capacity of oxidative phosphorylation (OXPHOS) in situ. This methodology is advantageous over measurements on isolated mitochondria since it: A) substantially reduces the overall tissue necessary, B) avoids selective isolation and assessment of mitochondrial subpopulations and, C) comprehensively examines function of all mitochondria within a muscle sample, independent of size, location, functional status, and in their natural intracellular position and assembly.

Blue native polyacrylamide gel electrophoresis (BN-PAGE) will be used to determine content and enzymatic activity of individual mitochondrial respiratory complexes.

Specific Techniques:

Determination of mitochondrial function using high-resolution respirometry of permeabilized muscle fibers.

Permeabilized skeletal muscle samples will be prepared for respirometry as described in (20). Briefly, 2-10 mg of freshly collected muscle sample will be washed in 1 mL ice-cold isolation medium (10 mM Ca-EGTA, 0.1 μM free calcium, 20 mM imidazole, 20 mM taurine, 49 mM K-MES, 3 mM K2HPO4, 9.5 mM MgCl2, 5.7 mM ATP, 15 mM phosphocreatine, 1 μM leupeptin, pH 7.1). Skeletal muscle strips will be separated to form thin muscle-fiber bundles, permeabilized in isolation medium containing 50 μg mL-1 saponin, at 4 °C, and washed several times in respiration medium (0.5 mM EGTA, 3

mM $MgCl_2$, 20 mM taurine, 10 mM KH_2PO_4, 20 mM HEPES, 1 g L^{-1} essentially fatty-acid free BSA, 60 mM K-lactobionate, 110 mM mannitol, 0.3 mM DTT, pH 7.1). Respiration medium will be added to the high-resolution Oxygraph 2k (OROBOROS INSTRUMENTS, Innsbruck, Austria), equilibrated to the required experimental temperature (37 °C) and oxygenated with atmospheric oxygen to ~600 μM O_2. The permeabilized muscle fiber bundle will be blotted dry, weighed and added to the chamber. Respiratory function of mitochondria and flux ratios will be determined using the following titration protocol (final concentrations and assessment in parenthesis). After assessment of resting respiration, the following reagents will be added: glutamate/malate (10mM/2mM; leak rate with complex I supported respiration); ADP (1-5 mM; state 3, complex I supported respiration; OXPHOS capacity C-I); cytochrome c (10 μM; integrity of the outer mitochondrial membrane); succinate (10 mM; state 3, complex II supported respiration; OXPHOS capacity C-I+II); oligomycin (2 μg mL^{-1}; inhibition of ATP synthase); stepwise titration with the uncoupler FCCP (0.5-5 μM; ETC capacity and limitation of OXPHOS relative to ETC by the phosphorylation system); rotenone (0.5μM; complex II-only supported respiration); antimycin A (2.5 μM; non-respiratory residual oxygen consumption). The respiratory control ratio (RCR), or OXPHOS capacity, will be determined by calculating the ratio of state 3 (with complex I + II substrates, and ADP) to state 4 (oligomycin-inhibited respiration); the ETC capacity will be determined using the ratio of uncoupled respiration to state 3 respiration. In addition to the calculation of flux control ratios, respiration will be related to mitochondrial DNA content (21) as well as citrate synthase activity (22).

Determination of respiratory complex content and activities using Blue Native Polyacrylamide Gel Electrophoresis (BN-PAGE).

For determination of the content and activity of the ETC complexes we will follow the protocol as described by Schagger et al. with some modification (23, 24) . Briefly, ~30 mg of muscle tissue will be homogenized in buffer 1 (20 mM MOPS, 440 mM sucrose, 1 mM EDTA and 0.5 mM PMSF, pH 7.2,) at 4°C. The homogenates will be centrifuged at 20,000 x g for 20 min. The pellet will be re-suspended in 80 μl of buffer 2 (1 M aminocaproic acid, 50 mM Bis-tris and 0.5 mM PMSF, pH 7.0). The membranes will be solubilized by the addition of 30 μl 10% n-dodecylmaltoside. Mitochondrial suspensions will be incubated on ice for 30 min and vortexed every 5 min, followed by ultracentrifugation for 25 min at 100,000 x g (Beckman, Optima LE-80K). The solubilized mitochondrial membrane proteins (in 5% w/v Coomassie brilliant blue G-250 and 1M aminocaproic acid) will be separated by BN-PAGE using a 3-12 % gradient gel. The anode buffer is comprised of 50 mM Bis-Tris, pH 7.0. The cathode buffer is comprised of 50 mM tricine, 15 mM Bis-Tris, and Coomassie brilliant blue G-250 (0.02% w/v), pH 7.0. Samples will be electrophoresed at 90 V for 20 min, and thereafter at 170 V for 2 h, at 4°C. For determination of the content of all of the respiratory complexes, gels will be blotted onto polyvinylidene fluoride membranes, which will then be probed with the MitoProfile Human Total OXPHOS Detection Kit (Mitosciences). For determination of enzymatic activity of respiratory complexes I, II, IV and V (complex III activity cannot be assessed using this technique), in-gel enzymatic colorimetric reactions will be performed. Gels will be incubated overnight at room temperature with the following solutions: Complex I: 2 mM Tris-HCl, pH 7.4, 0.1 mg/ml NADH, and 2.5

mg/ml NTB (nitrotetrazolium blue). Complex II: 4.5 mM EDTA, 10 mM KCN, 0.2 mM phenazine methasulfate, 84 mM succinic acid and 50 mM NTB in 1.5mM phosphate buffer, pH 7.4. Complex IV: 5 mg 3:30-Diamidobenzidine tetrahydrochloride (DAB) dissolved in 9 ml phosphate buffer (0.05 M, pH 7.4), 1 ml catalase (20 μg/ml), 10 mg cytochrome c, and 750 mg sucrose. Complex V: 35 mM Tris, 270 mM glycine, 14mM MgSO4, 0.2% Pb(NO3)2, and 8 mM ATP, pH 7.8. Gels will be washed in distilled water and the bands quantified by digital imaging using a CCD camera. Activity and content will be normalized to muscle weight, mitochondrial DNA content (21) as well as citrate synthase activity (22).

3. Titan analysis: The integrity and phosphorylation of Titan subunits of the sarcomere will be analyzed in Dr. Ottenheijm's laboratory at the University of Arizona. Dr. Ottenheijm's group has extensive experience making this unique measurement and will follow procedures as they have previously published.(25, 26)

Statistical Analysis:

We will use a mixed effects model with random intercept for each subject to evaluate the fixed effects of stimulation and fiber type on single-fiber force mechanics. For other dependent variables (i.e. mitochondrial respiration, RNA, protein quantification), we will test differences between the stimulated and unstimulated tissue samples using paired parametric T tests when the data meet the assumptions of parametric analysis, or non-parametric tests if the data are not normally distributed.

7. Possible Discomforts and Risks:

The risks of diaphragm biopsy include failure of the biopsy site to close, bleeding, phrenic nerve injury, and diaphragm dysfunction. The portion of the diaphragm from which the biopsy is obtained (6 mm by 20 mm section of tissue) will be isolated from the remaining muscle by surgical staples, prior to excision of the full-thickness biopsy sample. The site will also be reinforced with sutures, minimizing the risks of bleeding or failure of the biopsy site to close.

While human diaphragm biopsies are a relatively new model to study muscle, numerous workers have obtained non-therapeutic, experimental human diaphragm tissue samples during routine surgeries, as proposed in this work (27-35) and no complications were reported. We have recently completed a study in which two diaphragm samples were obtained from 5 subjects at the beginning and end of CT surgery, and there were no intra or postoperative complications associated with the diaphragm biopsies. The addition of indirect phrenic nerve stimulation to the protocol poses no significant risk to the subjects.

Respiratory testing (pulmonary function testing & maximum inspiratory pressure testing) may elicit transient fatigue or shortness of breath that resolves within several seconds of completion. Each training set typically lasts less than 15 seconds, and at least two to three minutes of rest will be provided between trials to minimize feelings of exertion, and perceived exertion will be monitored during training sessions.

This study may include risks that are unknown at this time.

8. Possible Benefits:

There are no direct benefits to the subjects for participating in this study, but our results will lead to a better understanding of how MV affects human diaphragm and may lead to improved clinical care for MV patients. The potential importance of optimizing diaphragmatic function prior to surgery was show by Hulzebos (2006). These researchers showed that 2 weeks of specific diaphragm training prior to coronary bypass surgery significantly reduced the incidence of post operative pneumonia and duration of hospitalization. The molecular basis of the improved post-operative diaphragmatic function following preoperative conditioning is unknown.

9. Conflict of Interest:

No real or potential conflict of interest exists for any investigator with regard to this research project.

Reference:

1. Siafakas NM, Vermeire P, Pride NB, Paoletti P, Gibson J, Howard P, Yernault JC, Decramer M, Higenbottam T, Postma DS, et al. Optimal assessment and management of chronic obstructive pulmonary disease (COPD). The European Respiratory Society Task Force. Eur Respir J. 1995;8(8):1398-420. Epub 1995/08/01. PubMed PMID: 7489808.
2. van Hees HW, van der Heijden HF, Ottenheijm CA, Heunks LM, Pigmans CJ, Verheugt FW, Brouwer RM, Dekhuijzen PN. Diaphragm single-fiber weakness and loss of myosin in congestive heart failure rats. American journal of physiology Heart and circulatory physiology. 2007;293(1):H819-28. Epub 2007/04/24. doi: 00085.2007 [pii] 10.1152/ajpheart.00085.2007. PubMed PMID: 17449557.
3. ATS/ERS Statement on respiratory muscle testing. Am J Respir Crit Care Med. 2002;166(4):518-624. Epub 2002/08/21. PubMed PMID: 12186831.
4. Kong KL, Cooper GM. Recovery of neuromuscular function and postoperative morbidity following blockade by atracurium, alcuronium and vecuronium. Anaesthesia. 1988;43(6):450-3. Epub 1988/06/01. PubMed PMID: 2900608.
5. Testelmans D, Maes K, Wouters P, Powers SK, Decramer M, Gayan-Ramirez G. Infusions of rocuronium and cisatracurium exert different effects on rat diaphragm function. Intensive Care Med. 2007;33(5):872-9. Epub 2007/03/16. doi: 10.1007/s00134-007-0584-4. PubMed PMID: 17361387.
6. Hutton P, Burchett KR, Madden AP. Comparison of recovery after neuromuscular blockade by atracurium or pancuronium. Br J Anaesth. 1988;60(1):36-42. Epub 1988/01/01. PubMed PMID: 3337792.
7. Day NS, Blake GJ, Standaert FG, Dretchen KL. Characterization of the train-of-four response in fast and slow muscles: effect of d-tubocurarine, pancuronium, and vecuronium. Anesthesiology. 1983;58(5):414-7. Epub 1983/05/01. PubMed PMID: 6132567.

8. Sprinkle JD, Takaro T, Scott SM. Phrenic Nerve Stimulation as a Complication of the Implantable Cardiac Pacemaker. Circulation. 1963;28(1):114-6.
9. Alyautdin RN, Buyanov VV, Fisenko VP, Lemina EJ, Muratov VK, Samoilov DN, Shorr VA. On some properties of a new steroid curare-like compound pipecurium bromide. Arzneimittelforschung. 1980;30(2a):355-7. Epub 1980/01/01. PubMed PMID: 6248077.
10. Moerer O, Baller C, Hinz J, Buscher H, Crozier TA. Neuromuscular effects of rapacuronium on the diaphragm and skeletal muscles in anaesthetized patients using cervical magnetic stimulation for stimulating the phrenic nerves. Eur J Anaesthesiol. 2002;19(12):883-7. PubMed PMID: 12510907.
11. DiMarco AF, Onders RP, Ignagni A, Kowalski KE, Mortimer JT. Phrenic nerve pacing via intramuscular diaphragm electrodes in tetraplegic subjects. Chest. 2005;127(2):671-8. PubMed PMID: 15706014.
12. Han YS, Proctor DN, Geiger PC, Sieck GC. Reserve capacity for ATP consumption during isometric contraction in human skeletal muscle fibers. J Appl Physiol. 2001;90(2):657-64. Epub 2001/02/13. PubMed PMID: 11160066.
13. Ferreira LF, Moylan JS, Stasko S, Smith JD, Campbell KS, Reid MB. Sphingomyelinase depresses force and calcium sensitivity of the contractile apparatus in mouse diaphragm muscle fibers. J Appl Physiol. 2012;112(9):1538-45. Epub 2012/03/01. doi: 10.1152/japplphysiol.01269.2011. PubMed PMID: 22362402; PMCID: 3362233.
14. Geiger PC, Cody MJ, Sieck GC. Force-calcium relationship depends on myosin heavy chain and troponin isoforms in rat diaphragm muscle fibers. J Appl Physiol. 1999;87(5):1894-900. Epub 1999/11/24. PubMed PMID: 10562634.
15. Gosselin LE, Johnson BD, Sieck GC. Age-related changes in diaphragm muscle contractile properties and myosin heavy chain isoforms. Am J Respir Crit Care Med. 1994;150(1):174-8. Epub 1994/07/01. PubMed PMID: 8025746.
16. Betters JL, Criswell DS, Shanely RA, Van Gammeren D, Falk D, Deruisseau KC, Deering M, Yimlamai T, Powers SK. Trolox attenuates mechanical ventilation-induced diaphragmatic dysfunction and proteolysis. Am J Respir Crit Care Med. 2004;170(11):1179-84. Epub 2004/09/18. doi: 10.1164/rccm.200407-939OC 200407-939OC [pii]. PubMed PMID: 15374845.
17. McClung JM, Kavazis AN, Whidden MA, DeRuisseau KC, Falk DJ, Criswell DS, Powers SK. Antioxidant administration attenuates mechanical ventilation-induced rat diaphragm muscle atrophy independent of protein kinase B (PKB Akt) signalling. The Journal of physiology. 2007;585(Pt 1):203-15. Epub 2007/10/06. doi: jphysiol.2007.141119 [pii] 10.1113/jphysiol.2007.141119. PubMed PMID: 17916612.
18. Kavazis AN, Talbert EE, Smuder AJ, Hudson MB, Nelson WB, Powers SK. Mechanical ventilation induces diaphragmatic mitochondrial dysfunction and increased oxidant production. Free Radic Biol Med. 2009;46(6):842-50. Epub 2009/02/03. doi: S0891-5849(09)00021-5 [pii] 10.1016/j.freeradbiomed.2009.01.002. PubMed PMID: 19185055.
19. Whidden MA, McClung JM, Falk DJ, Hudson MB, Smuder AJ, Nelson WB, Powers SK. Xanthine oxidase contributes to mechanical ventilation-induced diaphragmatic oxidative stress and contractile dysfunction. J Appl Physiol. 2009;106(2):385-94.

Epub 2008/11/01. doi: 91106.2008 [pii] 10.1152/japplphysiol.91106.2008. PubMed PMID: 18974366.
20. Kuznetsov AV, Veksler V, Gellerich FN, Saks V, Margreiter R, Kunz WS. Analysis of mitochondrial function in situ in permeabilized muscle fibers, tissues and cells. Nature protocols. 2008;3(6):965-76. Epub 2008/06/10. doi: nprot.2008.61 [pii] 10.1038/nprot.2008.61. PubMed PMID: 18536644.
21. Cormio A, Milella F, Marra M, Pala M, Lezza AM, Bonfigli AR, Franceschi C, Cantatore P, Gadaleta MN. Variations at the H-strand replication origins of mitochondrial DNA and mitochondrial DNA content in the blood of type 2 diabetes patients. Biochim Biophys Acta. 2009;1787(5):547-52. Epub 2009/04/07. doi: S0005-2728(09)00019-X [pii] 10.1016/j.bbabio.2009.01.008. PubMed PMID: 19344660.
22. Shepherd D, Garland PB. The kinetic properties of citrate synthase from rat liver mitochondria. Biochem J. 1969;114(3):597-610. Epub 1969/09/01. PubMed PMID: 5820645; PMCID: 1184933.
23. Schagger H, Cramer WA, von Jagow G. Analysis of molecular masses and oligomeric states of protein complexes by blue native electrophoresis and isolation of membrane protein complexes by two-dimensional native electrophoresis. Anal Biochem. 1994;217(2):220-30. Epub 1994/03/01. doi: S0003269784711122 [pii]. PubMed PMID: 8203750.
24. Wittig I, Braun HP, Schagger H. Blue native PAGE. Nat Protoc. 2006;1(1):418-28. Epub 2007/04/05. doi: nprot.2006.62 [pii] 10.1038/nprot.2006.62. PubMed PMID: 17406264.
25. Ottenheijm CA, Voermans NC, Hudson BD, Irving T, Stienen GJ, van Engelen BG, Granzier H. Titin-based stiffening of muscle fibers in Ehlers-Danlos Syndrome. J Appl Physiol. 2012;112(7):1157-65. Epub 2012/01/10. doi: 10.1152/japplphysiol.01166.2011. PubMed PMID: 22223454.
26. van Hees HW, Schellekens WJ, Andrade Acuna GL, Linkels M, Hafmans T, Ottenheijm CA, Granzier HL, Scheffer GJ, van der Hoeven JG, Dekhuijzen PN, Heunks LM. Titin and diaphragm dysfunction in mechanically ventilated rats. Intensive Care Med. 2012;38(4):702-9. Epub 2012/02/14. doi: 10.1007/s00134-012-2504-5. PubMed PMID: 22327561.
27. Ottenheijm CA, Heunks LM, Dekhuijzen RP. Diaphragm adaptations in patients with COPD. Respiratory research. 2008;9:12. Epub 2008/01/26. doi: 1465-9921-9-12 [pii] 10.1186/1465-9921-9-12. PubMed PMID: 18218129.
28. Ottenheijm CA, Jenniskens GJ, Geraedts MC, Hafmans T, Heunks LM, van Kuppevelt TH, Dekhuijzen PN. Diaphragm dysfunction in chronic obstructive pulmonary disease: a role for heparan sulphate? Eur Respir J. 2007;30(1):80-9. Epub 2007/03/30. doi: 09031936.00125106 [pii] 10.1183/09031936.00125106. PubMed PMID: 17392321.
29. Ottenheijm CA, Heunks LM, Hafmans T, van der Ven PF, Benoist C, Zhou H, Labeit S, Granzier HL, Dekhuijzen PN. Titin and diaphragm dysfunction in chronic obstructive pulmonary disease. Am J Respir Crit Care Med. 2006;173(5):527-34. Epub 2005/12/13. doi: 200507-1056OC [pii] 10.1164/rccm.200507-1056OC. PubMed PMID: 16339921.

30. Ottenheijm CA, Heunks LM, Li YP, Jin B, Minnaard R, van Hees HW, Dekhuijzen PN. Activation of the ubiquitin-proteasome pathway in the diaphragm in chronic obstructive pulmonary disease. Am J Respir Crit Care Med. 2006;174(9):997-1002. Epub 2006/08/19. doi: 200605-721OC [pii] 10.1164/rccm.200605-721OC. PubMed PMID: 16917114.
31. Ottenheijm CA, Heunks LM, Sieck GC, Zhan WZ, Jansen SM, Degens H, de Boo T, Dekhuijzen PN. Diaphragm dysfunction in chronic obstructive pulmonary disease. Am J Respir Crit Care Med. 2005;172(2):200-5. Epub 2005/04/26. doi: 200502-262OC [pii] 10.1164/rccm.200502-262OC. PubMed PMID: 15849324.
32. Levine S, Nguyen T, Taylor N, Friscia ME, Budak MT, Rothenberg P, Zhu J, Sachdeva R, Sonnad S, Kaiser LR, Rubinstein NA, Powers SK, Shrager JB. Rapid disuse atrophy of diaphragm fibers in mechanically ventilated humans. N Engl J Med. 2008;358(13):1327-35. Epub 2008/03/28. doi: 10.1056/NEJMoa070447. PubMed PMID: 18367735.
33. Moore AJ, Stubbings A, Swallow EB, Dusmet M, Goldstraw P, Porcher R, Moxham J, Polkey MI, Ferenczi MA. Passive properties of the diaphragm in COPD. J Appl Physiol. 2006;101(5):1400-5. PubMed PMID: 16840573.
34. Macgowan NA, Evans KG, Road JD, Reid WD. Diaphragm injury in individuals with airflow obstruction. Am J Respir Crit Care Med. 2001;163(7):1654-9. Epub 2001/06/13. PubMed PMID: 11401890.
35. Mercadier JJ, Schwartz K, Schiaffino S, Wisnewsky C, Ausoni S, Heimburger M, Marrash R, Pariente R, Aubier M. Myosin heavy chain gene expression changes in the diaphragm of patients with chronic lung hyperinflation. Am J Physiol. 1998;274(4 Pt 1):L527-34. Epub 1998/05/12. PubMed PMID: 9575870.
