# Supplementary material for: Intraoperative phrenic nerve stimulation to prevent diaphragm fiber weakness during thoracic surgery
Source: PLoS One. 2025 Apr 1;20(4):e0320936. doi: 10.1371/journal.pone.0320936 (PMC11961012; doi:10.1371/journal.pone.0320936)
Supplement: Attachment — Submitted filename: Review Response_PLos_IHS_final.docx [file pone.0320936.s014.docx]

**Review Comments to the Author

Please use the space provided to explain your answers to the questions above. You may also include additional comments for the author, including concerns about dual publication, research ethics, or publication ethics. (Please upload your review as an attachment if it exceeds 20,000 characters)**

**Reviewer #1: This is a report of a small cohort study. There are some deficiencies in the description of the statistical considerations of the study:**

**1. Sample size discussion is inadequate. What is the primary outcome of the study? What test statistic is being used? It appears that random effects models are being used in the analysis, yet there is no discussion of assumptions on the model that are necessary for computing sample size. Referencing a prior study is insufficient, as this study needs to stand alone. The Discussion should also include a sentence on whether the assumptions used in the sample size computation (variability etc.) were actually realized in the clinical trial.**

**2. Random effects models have assumptions that require validation of those assumptions. What diagnostics did you use to ensure the models are apt?**

**3. p<0.05 to declare significance is fine for the primary outcome, but there are other tests being done and there does not appear to be any adjustment for multiple testing. Of course the other tests may be exploratory, in which case p<0.05 really has no meaning. acknowledging that and declaring that a subsequent study with preplanned hypotheses should be conducted to confirm the observed association.**

RESPONSE TO COMMENTS #1, #2, and #3

We have added new information regarding sample size determination in the methods and discussion/limitations section. The sample size was determined for mitochondrial respiration and deemed sufficient to explore the effects on single fiber specific force based on our previous studies. This is now clarified in the manuscript (Page 14-15, excerpt below).

*Page 14-15: “The sample size for the trial was determined in advance of the study, based on our prior work examining intraoperative changes in diaphragm mitochondrial function (26). We did not have specific force data from type I and II fibers in this population to perform sample size calculations for the study. We proceeded to explore effects on single fiber function under the assumption that by measuring at least 6 fibers per side, but aiming to measure as many fibers as possible, the number of subjects included would be sufficient to detect specific force differences between sides based on published studies from our group testing human fiber contractile function, e.g., (1, 6, 27).”*

In this study, we used a ‘generalized linear mixed model,’ which includes both random and fixed effects. This is different from a ‘*random effects model’.*

The primary outcome for this component of the study was single fiber specific force. However, we perform several measurements that have biophysical relevance in each cell, and these are included here for completeness. Because these variables are available and informative, we performed statistical tests on them with the linear mixed model. The reviewer is treating those variables as a ‘clinical’ output. However, this was not our study design. It is true that the chances of type I statistical error increase as more variables are compared. But multiple comparison correction in the case of testing physiological and biophysical properties of the cells is relevant when multiple comparisons are being done for the same variable. Overall, our study is an exploratory trial, even for the primary outcome, and strict control of family wise error rate will be misleading with the exploratory variables. Complementary data provide insights into potential mechanisms, hence, we don’t consider the p<0.05 meaningless in that setting. However, most of the secondary outcome comparisons between groups were not statistically significant. Above all, it is important to consider that our study is not aiming to validate the use of our intervention in the clinical setting, which would have required separate control and treatment groups with assessments of clinical outcomes. Even in such a setting, taking an unnecessarily strict approach to multiple test correction can also be misleading (PMID: 27106412). We have modified the manuscript accordingly (excerpt below).

Page 15: *“The individual fiber data were used to compare effects of stimulation on contractile function via a generalized linear mixed model (SAS 9.4), which accounted for the repeated and correlated measures contained with each subject’s paired stimulated and control samples. The primary exploratory outcome of interest in the study was single fiber specific force. The linear mixed model approach provides superior statistical power and confidence to grouping the data and analyzing with simpler approaches, such as t-tests or ANOVA. Fixed effects included stimulation and fiber type, while the subject was treated as a random intercept effect. We confirmed homoscedasticity and normality by visual inspection of residuals. The assumption of randomness is based on inclusion of all patients that met criteria and consented to participate in the study.”*

*Page 16: “We considered statistical significance when p<0.05 and did not correct for multiple tests of secondary endpoints because these were performed to gain insights into potential biophysical and physiological mechanisms underlying the primary endpoint of specific force. In this context, p < 0.05 is meant to represent potential physiological or biophysical relevance and not clinical significance.”*

*Page 19: “We confirmed the statistical difference in the primary endpoint (specific force) with a Mann-Whitney test (p = 0.0290; median: control 91 kN/m^2^, STIM 101 kN/m^2^).… In fast fibers, we did not detect significant differences between stimulated and non-stimulated hemidiaphragms for any of the variables examined (Fig 2E-H and S2; also confirmed by non-parametric Mann-Whitney test; p = 0.413)”*

We agree that we must acknowledge the exploratory nature and have added a statement as suggested by the reviewer. The relevant sentence is copied below and this is also addressed in the material on page 15 (see example above)

*Page 5: “The objective of the current study was to explore whether brief bouts of intermittent electrical phrenic nerve stimulation attenuate diaphragm slow and fast fiber weakness…”*

**Reviewer #2: GENERAL COMMENTS**

**Bresciani et al assessed the effects of unilateral phrenic nerve stimulation during cardiothoracic surgery on diaphragm muscle fiber function. This is huge work, with a very complex population and very complicated collection of diaphragm biopsies. I provided point-by-point comments below. Some of them are already described by the authors as limitations. However, I consider them to be very important points that need to be considered while reviewing/considering the manuscript for publication. I hope these comments help you improve the quality of the manuscript.
There some differences between the clinical trials registration and the results reported here.

ABSTRACT
Please, clarify details of the stimulation (e.g., duration of each stimulation and etc)?**
We have added the details to the abstract

*“Unilateral phrenic twitch stimulation (twitch duration 1.5 ms, frequency 0.5 Hz, current 2x the motor threshold, max 25 mA) was applied for one minute, every 30 minutes during cardiothoracic surgery.”*

**MAIN TEXT
It is not clear why the focus of this study was patients with normal lung function/inspiratory muscle strength. The question would be much more relevant in high-risk populations, such as patients with lung diseases.**

The issue of relevance is a matter of debate. We do not have evidence that patients with lung diseases are more susceptible to ventilator-induced diaphragm dysfunction during open chest procedures than the population we studied. We consider that the population targeted in our study is more relevant because the number of open-chest surgeries in these individuals (valve repair, CABG, etc) far exceed the number performed in those with lung diseases that require a open-chest procedure.

**The study used unilateral phrenic nerve stimulation and considered the other side as an internal control. Unilateral phrenic nerve stimulation can sometimes cause both hemidiaphragms to contract. This phenomenon occurs due to the presence of cross-innervation or central connections between the motor neurons controlling the diaphragm. When one phrenic nerve is stimulated, it can lead to a bilateral response, causing both hemidiaphragms to contract, although the effect is typically stronger on the side directly stimulated it imposes a limitation on what is considered the “control hemidiaphragm” for the study. This may underestimate the effect of the intervention, which could affect the conclusions that, for instance, only one type of fiber was found to be different.**

We agree that unilateral stimulation may have underestimated the potential benefits of diaphragm stimulation. This topic has been debated publicly by our groups and are now explicitly mentioned and cited in the manuscript.

Page 30: *“Moreover, supramaximal stimulation and contraction of a hemidiaphragm applies slight lengthening forces upon the contra-lateral unstimulated side. Diaphragm stretches might be enough to blunt the detrimental effects of MV and inactivity,(61) and the unstimulated side may not have represented a pure “inactivity” control. In this case, our results would underestimate the benefits of intermittent phrenic stimulation.”*

**Please, clarify whether or not neuromuscular block was used at any point in any patients for the surgical procedure.**

We have added new information regarding the use of neuromuscular block for induction in nearly all patients and maintenance in a small subset of patients. The anesthesiology team was aware of the study procedures and tailored their anesthesiology regimen to minimize neuromuscular block whenever possible. The presence of neuromuscular block may have underestimated the benefits of stimulation. However, the inclusion of these patients enhances ecological validity of the findings to support completion of a trial with bilateral stimulation during open chest surgery.

**If the protocol was extended to include patients with lung transplant, why reporting partial data only here? and from patients with preserved MIP? The results from patients post-lung transplant would be much more interesting from a clinical perspective.**

We agree that intraoperative stimulation during lung transplant is interesting. That was the rationale for expanding the protocol and obtaining additional data with the goal of collecting pilot data and eventually studying enough patients to resolve the different populations. However, we were able to enroll only two participants with lung transplants on the project. For one subject, the investigator conducting single fiber experiments was not available. Since single fiber data were only available for one participant, we elected to report these separately in abstract form (<https://faseb.onlinelibrary.wiley.com/doi/full/10.1096/fasebj.2022.36.S1.R4425>). However, we consider that the population included in our study is relevant because inspiratory muscle weakness affects post-operative outcomes for open-chest surgeries for the conditions in our study (valve repair, CABG, etc) and the number of these procedures per year far exceed that for lung or heart transplants. But considering the latter are much longer procedures, we agree that phrenic stimulation may have a bigger impact on VIDD protection. In this context, the key aspect is surgery duration and not necessarily the underlying disease conditions.

**Line 164. I am not sure I understand the parameters for the stimulation correctly. Please, clarify: each stimulation was continuously applied for 1 minute? If that is correct, I do not understand the rationale for these settings. Each inspiratory effort last approximately 0.8 to 1.2 seconds. I would expect a stimulation targeted to protect the diaphragm to have such a physiological range. If the stimulation lasted one minute, this does not resemble the way the diaphragm works normally. Or did the stimulation last 1.5 ms? If this is the case, this is the complete opposite and similarly does not resemble how the diaphragm works normally. Please, clarify the parameters in the text. I suggest this is addressed in the limitations section and please describe the rationale for this choice.**

We have added details of the stimulation parameters in the text. The stimulation lasted 1.5 ms and was repeated 30x within one minute. Indeed, this does not reflect the normal contraction pattern of the diaphragm with phrenic firing frequency of 4-9 Hz and breathing frequency of 10-20 breaths/min. Our goal was to elicit contractions to activate diaphragm metabolic and mechano-transduction pathways that are inactive during mechanical ventilation while minimizing patient movement, bilateral effects, and prolonged interruption/interference with the surgical procedure.

Page 8: *“Twitch stimulation parameters included a 1.5 ms pulse width and contraction frequency of 0.5 Hz (one twitch every 2 seconds) for a total of 1 min duration. Atrial output was 0 mA, and ventricular output was set at twice the visible twitch threshold (up to the device maximum current of 25mA), which ensured a vigorous hemidiaphragm contraction. We delivered the first stimulation from the time the phrenic nerves were initially exposed and repeated the protocol every 30 min until completion of the procedure. We opted for this protocol to minimize patient movement with stronger tetanic contractions, bilateral impact of stimulations, and interruption of the surgical procedure.”*

**Sample size was based on prior work about mitochondrial function. The effect size or the magnitude of the difference between the two groups used for the sample size calculation is not described. Moreover, mitochondrial function is not a primary outcome in the study. Please, clarify the rationale for this approach and why not calculate the sample size based on differences expected for the primary outcomes of the study?**

The sample size was projected from preliminary data presented in R01AR072328, which included specific aims for mitochondrial function (Aim 1), single fiber diaphragm contractility (Aim 2), and catabolic pathway activation (Aim 3). The results from Aims 1 and 3 have been published separately. We have added new information regarding sample size determination in the methods and discussion/limitations section. The sample size was determined for mitochondrial respiration and deemed sufficient to explore the effects on single fiber specific force based on our previous studies. This is now clarified in the manuscript (Page 14-15, excerpt below).

*Page 14-15: “The sample size for the trial was determined in advance of the study, based on our prior work examining intraoperative changes in diaphragm mitochondrial function (26). We did not have specific force data from type I and II fibers in this population to perform sample size calculations for the study. We proceeded to explore effects on single fiber function under the assumption that by measuring at least 6 fibers per side, but aiming to measure as many fibers as possible, the number of subjects included would be sufficient to detect specific force differences between sides based on published studies from our group testing human fiber contractile function, e.g., (1, 6, 27).”*

**Calculated sample size was n=20 but 25 patients were included. Please, justify. Then, data is available from 19 patients, which is less than the calculated sample size. Looking at the first clinical trial registration (**[**https://clinicaltrials.gov/study/NCT03303040?tab=history&a=1#version-content-panel**](https://clinicaltrials.gov/study/NCT03303040?tab=history&a=1#version-content-panel)**), estimated enrollment was 54 and not all outcomes described in the clinical trials registration are reported here. Please, clarify.**

The enrollment of 54 participants projected in the original clinicaltrials.gov entry reflected the potential that individuals might be enrolled into the study but might subsequently drop out in advance of surgery or not complete the specified protocol for unrelated clinical reasons. The sample size projection identified 20 completers would be needed; we enrolled 25 patients, and 21 completed the study. Nineteen of the completed participants underwent conventional open cardiac surgery, and the remaining two received lung transplants. Single fiber data was only available from one lung transplant patient. Since single fiber data were only available for one participant, we elected to report these separately in abstract form (<https://faseb.onlinelibrary.wiley.com/doi/full/10.1096/fasebj.2022.36.S1.R4425>).

Several other outcomes from this study were published separately in Mankowski et al, 2024 (<https://pubmed.ncbi.nlm.nih.gov/37237876/>) and other outcomes were abandoned due to limited sample availability for analyses.

**How was the intensity of the stimulation set since it was not the same for all? Based on the strength of the diaphragm contraction? The effect of the stimulation intensity and the effort generated by the diaphragm during each contraction caused by the stimulation could significantly affect the study results.**

The intensity of the stimulation was twice the motor threshold of the diaphragm, up to a maximum of 25 mA. The motor threshold of the stimulated hemidiaphragm was confirmed visually by the study personnel delivering the stimulation and the surgeon. We have modified the methods to clarify this aspect.

**The authors discuss that “However, synchronization of phrenic stimulation and MV-delivered lung inflation is not necessary (or physiologically relevant) during open-chest thoracic surgery. The clinical environment did not permit us to make quantification of force generation, but we could clearly observe vigorous contractions of the stimulated side. The optimal parameters and timing of phrenic stimulation remains unresolved.” However, studies have shown that, during mechanical ventilation, the timing (i.e., synchronous or dyssynchronous) type of contraction (e.g., concentric vs eccentric) and the effort generated by the diaphragm (e.g., large vs small effort) all can affect whether it will develop injury or not. These factors can influence diaphragm function independently or in combination.**

The reviewer is correct. However, these factors are in effect during mechanical ventilation with a *closed chest*. The sternotomy and spreading of rib cage create a physical dissociation of lungs and chest wall/diaphragm mechanics. Pressure in the intrapleural space equilibrates with ambient pressure upon sternotomy and the ensuing ‘pneumothorax’ such that contractions of inspiratory muscles cannot cause lung inflation. Likewise, inflation/deflation of the lungs by the mechanical ventilator during open chest surgery would have no direct effect on the diaphragm or other respiratory muscles. Therefore, we maintained the original text in the manuscript as it focuses on the situation encountered during an open-chest surgery.
